# Ecological drivers of *Clostridioides difficile* disease severity

**DOI:** 10.1128/iai.00296-26

**Published:** 2026-10-15

**Authors:** Rochelle C. Glover, Ana P. R. Catelli, Joseph P. Zackular

**Affiliations:** 1 Department of Pathology and Laboratory Medicine, Children’s Hospital of Philadelphia 548800, Philadelphia, Pennsylvania, USA; 2 The Center for Microbial Medicine, Children’s Hospital of Philadelphia 6567, Philadelphia, Pennsylvania, USA; 3 Department of Pathology and Laboratory Medicine, Perelman School of Medicine, University of Pennsylvania 14640, Philadelphia, Pennsylvania, USA; University of Pittsburgh, Pittsburgh, Pennsylvania, USA

**Keywords:** *Clostridioides difficile*, toxins, immune response, microbiome, ecology

## Abstract

*Clostridioides difficile* infection (CDI) is a common cause of antibiotic-associated diarrhea and arises from disruption of the resident microbiota in the gastrointestinal tract. Despite being a major cause of morbidity and mortality worldwide, CDI presents as a wide spectrum of clinical manifestations, ranging from completely asymptomatic infection to severe and fulminant colitis. Recent research has revealed that CDI disease severity is influenced by the complex ecosystem of the gut, involving interactions between *C. difficile*, the host, and the resident gut microbiota. This review will summarize the current state of the field in understanding how the balance of interactions in the gut alters CDI disease severity using clinical data and mouse models of infection. In addition, external factors influencing these interactions, such as diet and pharmaceutical drugs, will be discussed. Ultimately, a comprehensive understanding of the ecological factors influencing patient outcome will be critical for the design of novel therapeutics for primary and recurrent CDI.

## INTRODUCTION

The gastrointestinal (GI) tract of mammals is a dynamic ecosystem shaped by interactions between commensal microbes and the host. The ecological landscape of the GI tract plays a critical role in determining susceptibility to and severity of enteric infectious diseases. This relationship is particularly evident with *Clostridioides difficile* infection (CDI), which is one of the most common healthcare-associated infections in industrialized countries. In the United States, CDI cases surpass 500,000 annually, with up to 30,000 associated deaths (1). CDI arises through loss of colonization resistance by the host gut microbiome, most commonly through antibiotic treatment. *C. difficile* is a Gram-positive, spore-forming, obligate anaerobe and produces two large exotoxins, TcdA and TcdB, which cause colonic inflammation and damage to intestinal epithelial cells (IECs), leading to the main symptoms of disease, including diarrhea, pseudomembranous colitis, and toxin megacolon in severe cases. The only available treatment for a primary CDI is antibiotic therapy; however, this leads to high rates of recurrence due to prolonged dysbiosis.

**Fig 1:**
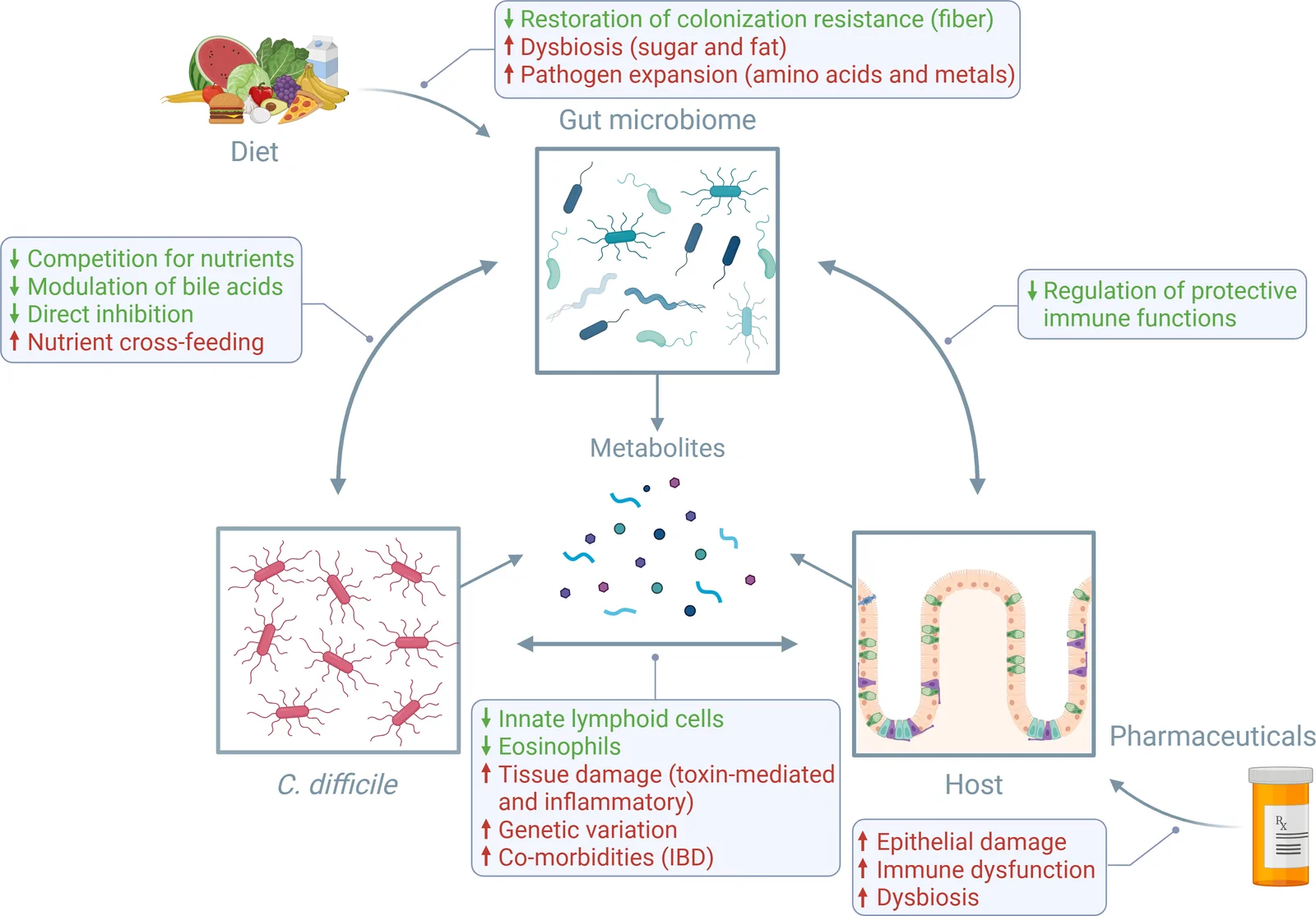
Ecological interactions in the GI tract during CDI. Ecological interactions between the gut microbiome, the host, *C. difficile*, and the metabolites produced by each determine the disease severity of CDI. Interactions that improve CDI outcome (green text, downward arrow) and worsen disease severity (red text, upward arrow) are depicted. External factors, such as diet and pharmaceuticals, influence CDI severity through modulation of these interactions.

Despite being a major cause of morbidity and mortality, clinical manifestation of CDI can vary markedly from patient to patient, with some displaying mild illness, while others develop severe disease (2). The factors shaping susceptibility to infection, severity of disease, and outcomes of infection remain incompletely defined; however, significant progress in deepening our knowledge of this complex disease has been made over the past two decades. In this mini-review, we will cover recent advances in understanding factors shaping CDI, with a focus on complex interactions between the host, microbiota, and pathogen (Fig. 1).

## HOST FACTORS CONTRIBUTING TO DISEASE SEVERITY

### Immune response to CDI

The immune response to *C. difficile* can both protect from disease and promote intestinal pathology. TcdA and TcdB activate pattern-recognition receptors (PRRs) to induce production of pro-inflammatory cytokines and chemokines, which promote a robust inflammatory response and recruit immune cells to the site of infection (3). Neutrophils are critical for restricting dissemination of commensal organisms through the damaged gut barrier and are, therefore, required for protection from severe complications of CDI, including secondary sepsis. Thus, ablation of neutrophils in mice increases mortality when challenged with *C. difficile* (4, 5). However, high abundances of neutrophils can also lead to tissue damage due to release of cytotoxic compounds (6). In CDI patients, blood leukocyte and neutrophil concentrations are highly correlated with worse disease outcomes (7–11). Two recent studies found that *C. difficile* infection of mice induced subpopulations of neutrophils in the colonic lamina propria that expressed increased activation and degranulation markers and were responsible for heightened IEC damage (12, 13). Together, these findings highlight the importance of a balanced neutrophil response during CDI that limits immunopathology while maintaining effective control of infection.

**Fig 2:**
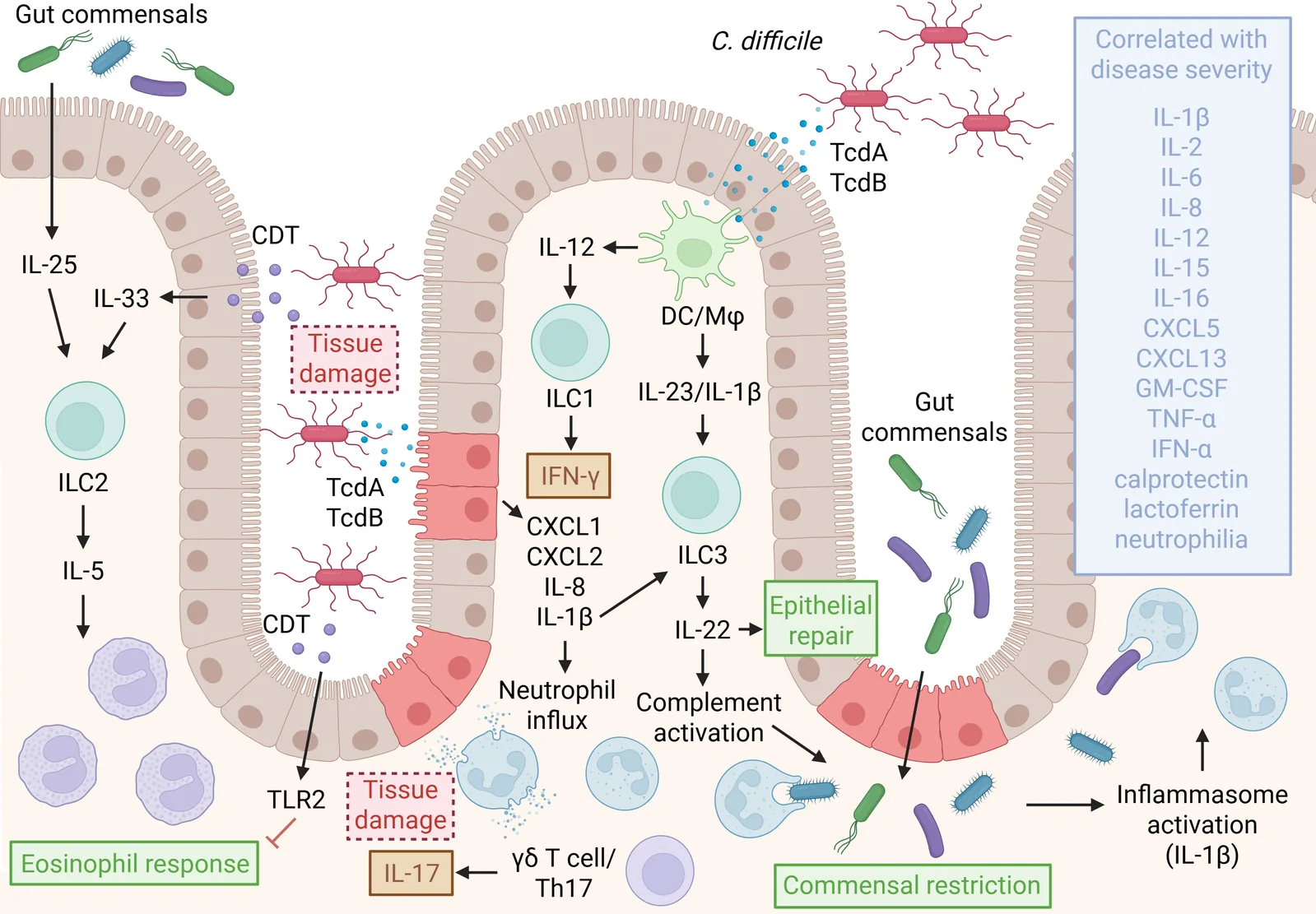
Contributions of the immune system to CDI severity. A balance of protective (green solid boxes) and pathogenic (red dashed boxes) immune responses influences the outcome of infection. *C. difficile* toxins (TcdA, TcdB, and CDT) activate pro-inflammatory pathways in epithelial and immune cells, inducing production of cytokines and chemokines. The cytokines IL-25, IL-33, IL-5, IL-12, IL-23, and IL-1β promote protective ILC and eosinophil responses. The chemokines CXCL1, CXCL2, and IL-8 recruit neutrophils to the site of infection, which are required for protection against CDI but also contribute to tissue damage. Toxin- and neutrophil-mediated epithelial damage allows commensal microbes to translocate across the intestinal barrier, which must be restricted by recruited immune cells. Certain cytokines, such as IFN-γ produced by ILC1 and IL-17 produced by T cells (orange solid boxes), can be both protective and pathogenic in different contexts. Immune factors associated with severe disease in CDI patients are shown in the light blue box.

Additional inflammatory mediators are associated with severe disease and mortality in CDI patients (Fig. 2) (11, 14–21). While some of these markers are likely associated with neutrophil-driven intestinal damage, others may be a result of additional inflammatory pathways. *C. difficile* activates inflammasomes in epithelial and immune cells via toxins, S-layer proteins (SLPs), and flagellin (22–25). Notably, inflammasome activation and cell-death pathways are influenced by both the concentration and cellular target of *C. difficile* toxins, with responses varying across cell types (26, 27). ASC deficiency reduces pathology in response to *C. difficile* toxins in a mouse ileal loop model, suggesting that inflammasome activation contributes to disease pathogenesis (25). However, ASC-deficient mice exhibit increased disease severity during oral challenge with *C. difficile*, suggesting that inflammasome activation can play differential roles depending on context (28). Additionally, production of IL-17, IL-23, CCL3 (MIP-1α), and macrophage migration inhibitory factor (MIF) was shown to contribute to pathogenic inflammation in mouse models of CDI (29–33).

Despite the strong association of the innate immune response with increased disease severity, beneficial immune functions have been shown to be equally as important for protecting against severe manifestations of CDI. TLR4 and TLR5 play a protective role during *C. difficile* challenge in mice. SLPs from *C. difficile* activate dendritic cells via TLR4, inducing DC maturation and induction of Th1/Th17-promoting cytokines. TLR4- and MyD88-deficient mice experience more severe disease and an inability to develop antibody responses to SLPs, suggesting that recognition of SLPs by TLR4 may play a protective role *in vivo* (34). TLR5 stimulation protects mice from severe CDI by reducing IEC apoptosis and increasing expression of antimicrobial peptides (35).

Protective immune responses are critical for preventing secondary complications from infection, including sepsis, induced by toxin-mediated epithelial damage. The cytosolic PRR NOD1 is activated during CDI and is required for the induction of a protective neutrophil response that restricts translocating commensal microbes (4). These disseminating microbes also activate inflammasomes in the underlying lamina propria, which promotes clearance through recruitment of neutrophils (28). Additionally, IL-22 production was found to be critical for restricting commensal dissemination, particularly *Enterobacteriaceae*, through activation of complement component C3 and increased phagocytosis of circulating bacteria (36).

Recent evidence has revealed an important role for type 2 immune responses in protection from severe CDI. Up to 20% of *C. difficile* strains encode for a third toxin, *C. difficile* transferase (CDT), or binary toxin. CDT induces production of IL-33, which drives activation of a protective type 2 innate lymphoid cell (ILC2) response (37). Subsequent production of IL-5 by ILC2s reduces disease severity and is associated with increased proportions of eosinophils in the colonic lamina propria (38). Indeed, reduced eosinophils or IL-33 signaling in CDI patients is correlated with increased disease severity (37, 39). Interestingly, despite the CDT-dependent induction of the IL-33/ILC2 pathway, CDT also antagonizes protective eosinophilic responses by direct eosinophil killing and activation of a pathogenic TLR2 response that suppresses eosinophil production (40). Other innate lymphoid cell types ILC1 and ILC3 also play an important role in reducing CDI disease severity through production of IL-22, IFN-γ, and GM-CSF (41–43). Collectively, these studies indicate a clear role for eosinophils and ILCs in protecting against morbidity and mortality caused by CDI.

*C. difficile* toxins, specifically TcdB, have been shown to have systemic effects beyond the gastrointestinal tract, causing peripheral organ damage that drastically increases disease severity (44). Blockade of systemic toxin burdens through the production of toxin-specific antibodies in serum is sufficient to completely ameliorate morbidity in a mouse model of severe CDI (45). Indeed, CDI patients with higher circulating anti-toxin antibodies are more likely to be protected from the development of symptoms and infection recurrence (46–50). However, many people fail to develop a protective immune response, which was recently shown to be due to the inhibition of germinal center and CD4^+^ T cell responses by systemic TcdB activity (51, 52). Development of future anti-toxin therapies should consider both the local and systemic effects of toxin activity to lower disease severity and promote the development of a protective immune response to *C. difficile*.

### Enteric nervous system responses and neurogenic inflammation

Emerging research indicates an important role of the nervous system during *C. difficile* pathogenesis. *C. difficile* toxins target enteric glial cells, neurons, and pericytes, causing cytopathic effects, cell death, and secretion of neurotransmitters and cytokines (53–61). While these effects were initially observed over 30 years ago (62, 63), more recent studies have defined their role in *C. difficile* pathogenesis. Blockade of toxin-mediated effects on enteric nervous system cells, including secretion of neurotransmitters (Substance P, CGRP, S100B, and norepinephrine) and activation of neuronal signaling receptors (adenosine receptor A2B, alpha 2 adrenergic receptor), reduced CDI disease severity in mouse models (53, 61, 64, 65). Additionally, *in vitro* studies demonstrated that the effects of TcdB on enteric glial cells are enhanced in the presence of the cytokines TNF-α and IFN-γ, suggesting possible synergy between the pro-inflammatory effects and neuronal targeting of *C. difficile* toxins (57, 66). Overall, these data suggest that the degree to which a patient experiences neurogenic inflammation may be an important contributor to CDI disease severity.

### Co-morbidities with CDI

It is well established that co-morbidities which affect immune function are associated with the development of severe disease. For example, old age is one of the major risk factors for poor outcomes, likely due to insufficient induction of protective immune responses (8, 67–69). Similarly, cancer patients undergoing chemotherapy and solid organ transplant patients experience significant immunosuppression and are at risk for increased mortality (70–72). In contrast, patients with conditions that increase intestinal inflammation, such as inflammatory bowel disease (IBD), are also at risk for severe manifestations of CDI (73–76). Despite these correlations being well-documented, the mechanisms of enhanced virulence in these altered gut ecosystems are unknown. Some data suggest that alterations of the gut microbiota and intestinal metabolites, such as bile acids, butyrate, and sugars, may enhance *C. difficile* fitness in IBD patients (77–84). Other studies have shown that pathogenic Th17 cells may contribute to disease severity of CDI specifically in the context of IBD (82, 85, 86). Interestingly, IL-17-producing T cells have been shown to be part of a beneficial adaptive immune response to CDI under different contexts, demonstrating that the balance between protective and pathogenic immune responses can be shifted by changes in gut ecology (34, 87–91). Additionally, cystic fibrosis patients exhibit high rates of asymptomatic *C. difficile* colonization which rarely progresses to disease despite other risk factors for CDI development such as high antibiotic use (92, 93). This suggests that alterations to the gut environment in cystic fibrosis patients may affect susceptibility to CDI. Overall, while associations with co-morbidities make understanding drivers of disease pathogenesis complicated, more work is warranted in exploring CDI in the context of complex co-morbidities.

### Host metabolism

Recent work has shown that host metabolic pathways can impact CDI progression through direct cross-feeding of metabolites to *C. difficile* or alterations of the gut microbiota. One study demonstrated that host aldose reductase, an enzyme that produces sorbitol, is upregulated during CDI and that *C. difficile* consumption of sorbitol increases fitness in the gut (94). Aldose reductase expression and sorbitol concentration were also increased under conditions of hyperglycemia, leading to increased *C. difficile* bacterial burdens (94). These data suggest that differences in aldose reductase activity and blood glucose levels can alter the severity of CDI. *C. difficile* infection of mice also induces glycolytic metabolism in the colon during the acute phase of infection, followed by a switch to oxidative phosphorylation during the recovery phase (95). Interestingly, inhibition of glycolysis during infection reduced disease severity by maintaining oxidative metabolism, which enhances epithelial tight junctions and tissue repair (95). Collectively, these data demonstrate a powerful influence of host cell metabolism over disease outcome and prompt further studies on non-microbial driven metabolism in the gut during CDI.

## *C. DIFFICILE* PATHOGENESIS

### Genetic variation of *C. difficile* strains

The public health burden of *C. difficile* grew rapidly in the mid-2000s with the emergence of the epidemic strain NAP1/BI/027 (PCR ribotype 027) which spread globally (96, 97). Since then, multiple studies have linked NAP1/BI/027 strains with not only increased rates of CDI but also increased disease severity and mortality (11, 21, 98–101). Genomic analysis of NAP1/BI/027 strains revealed several genetic changes, which were hypothesized to contribute to the emergence of these strains: (i) presence of genes encoding binary toxin; (ii) truncation of *tcdC*, a gene encoding a repressor of toxin synthesis; and (iii) mutations in *gyrA* that confer increased fluoroquinolone resistance (97). Indeed, studies have found that the presence of binary toxin and *tcdC* mutations, rather than the ribotype, predicts disease severity or mortality in CDI patients, as multiple other ribotypes carrying these genetic variants have since been found (7, 102–107). Additionally, NAP1/BI/027 strains can exhibit considerable pathogenic variability in animal models, with some strains demonstrating complete avirulence (108).

Experiments in animal models of CDI have demonstrated that *tcdC* mutations and binary toxin expression enhance *C. difficile* virulence *in vivo* (109–112). The role of binary toxin in *C. difficile* pathogenesis is still being elucidated, but several potential mechanisms have been described. Binary toxin increases pathogenic inflammation during CDI by activation of the TLR2/6 heterodimer and subsequent NF-κB and MAPK signaling (40, 113). Activation of mucosal-associated invariant T (MAIT) cells and skewing to a cytotoxic phenotype by binary toxin may also contribute to *C. difficile* pathogenesis (114). Finally, adherence of *C. difficile* to host cells is enhanced by binary toxin via the formation of microtubule-based protrusions on the surface of IECs as well as through the promotion of biofilm formation on intestinal mucosa (115–117). While more studies are needed for a complete picture of the role of binary toxin, it is clear that the expression of this virulence factor contributes to CDI disease severity.

Other genetic variations among *C. difficile* strains contribute to pathogenesis. The pathogenicity locus (PaLoc) encoding TcdA, TcdB, and other factors involved in toxin expression and export is an area of positive selection in the *C. difficile* genome and has undergone extensive divergence among *C. difficile* strains. In particular, *tcdB* contains highly diverse regions and has been divided into subtypes based on sequence similarity (118–120). Importantly, these TcdB subtypes have different cytotoxicity profiles *in vitro* (121–125). More work is needed to determine whether TcdB subtypes drive differences in disease severity *in vivo*; however, the recent finding that certain TcdB variants have altered host cell receptor tropism certainly suggests that TcdB diversity plays a role in *C. difficile*-host interactions (126, 127). Outside of toxins, genetic variation in other virulence factors, such as SLPs and flagella, has been implicated in alterations in immune system activation, sporulation, and toxin production (122, 128–130).

### Toxin regulation

The main drivers of *C. difficile* pathogenesis are the toxins TcdA and TcdB, and some studies suggest that fecal toxin levels in CDI patients correlate with disease (131–133). Therefore, research on factors that control toxin expression by *C. difficile* is critical for understanding how toxin levels change from patient to patient. TcdA and TcdB are regulated by TcdR, a sigma factor that activates *tcdA* and *tcdB* transcription. The expression of these genes is largely regulated by TcdR levels, rather than post-translational modifications of TcdR. The *tcdR* promoter is directly and indirectly controlled by over 10 different transcriptional regulators in *C. difficile* that sense the metabolic state of the cell and respond to changes in carbon source availability (sugars and amino acids), redox state, gut metabolites (short-chain fatty acids, SCFAs), and sporulation signals (134). Most of the studies on *C. difficile* toxin regulation focus on the contributions of a single regulator during *in vitro* conditions; it is unknown how the hierarchy of overlapping signals in the gut contributes to differences in toxin production *in vivo*. This is further complicated by the fact that regulators controlling *tcdR* transcription also affect the expression of critical metabolic pathways, so genetic ablation can often have pleiotropic effects outside of toxin synthesis. However, it is clear that the metabolic state of the gut has a heavy influence on *C. difficile* toxin production, and factors such as host and microbial metabolism, as discussed elsewhere in this mini-review, are likely to influence disease severity through effects on toxin expression. Additionally, toxin expression has recently been found to be heterogeneous within a population of *C. difficile* cells, with only a subset of cells expressing high levels of toxin (135, 136). The regulation of heterogeneous toxin expression is still being elucidated but may play an important role in shaping observed toxin titers during infection and, therefore, disease severity.

## INFLUENCE OF THE MICROBIOME

**Fig 3:**
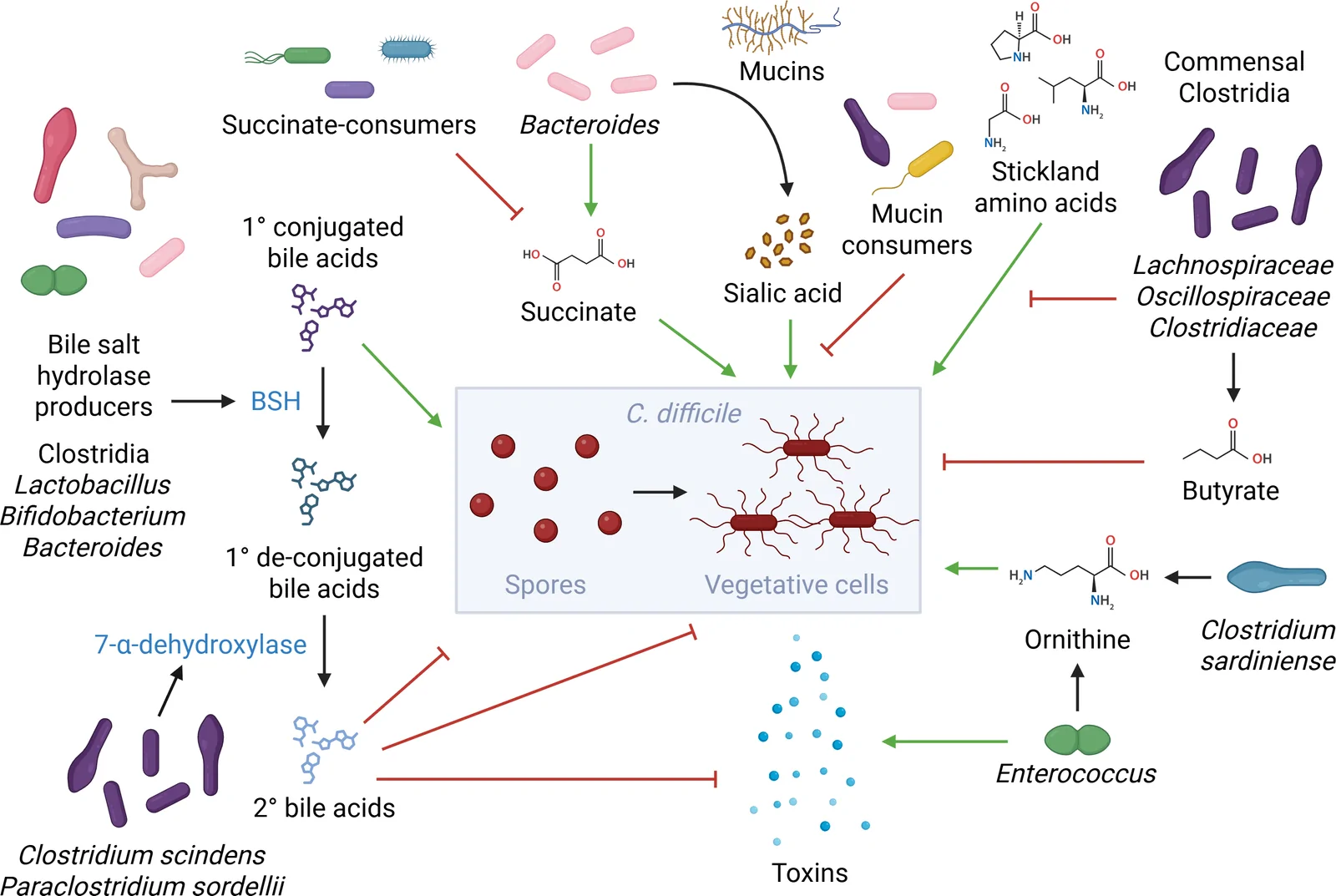
Influence of microbial-derived metabolites on *C. difficile* fitness. Metabolites produced by the gut microbiome both enhance (green arrows) and reduce (red blunt arrows) *C. difficile* fitness in the GI tract. Certain secondary bile acids produced through the action of microbial enzymes inhibit spore germination, vegetative cell growth, and toxin activity, while specific primary conjugated bile acids serve as germination signals. Production of the SCFA butyrate inhibits *C. difficile* growth. Succinate, sialic acid, and ornithine produced by the gut microbiome aid in *C. difficile* expansion. Stickland amino acids serve as a critical carbon and energy source for *C. difficile* in the gut, and consumption of these amino acids by commensal Clostridia restricts pathogen growth. Dysbiosis shifts the composition of the gut microbiome toward a metabolic state that promotes *C. difficile* pathogenesis.

The gut microbiome plays a critically important role in CDI pathogenesis. It has long been appreciated that gut microbes confer colonization resistance against *C. difficile*, as CDI is largely associated with antibiotic use. However, specific mechanisms of colonization resistance have only been recently elucidated. In addition, there is a growing body of literature demonstrating synergistic interactions in the gut microbiome that enhance *C. difficile* virulence. Overall, the balance of positive and negative microbe-microbe interactions in the gut influences the outcome of disease (Fig. 3).

### Bile acid metabolism

One of the most commonly proposed mechanisms of colonization resistance is the modulation of the intestinal bile acid pool by commensals. Bile acids are synthesized by the host as primary bile acids and conjugated to the amino acids taurine and glycine. After secretion into the gut, bile acids are modified by the microbiome through deconjugation of amino acids by bile salt hydrolases (BSHs) and chemical modification into secondary bile acids (137). Antibiotic treatment reduces taxa that perform these critical functions, shifting the bile acid pool toward mainly primary conjugated bile acids, some of which (particularly cholate and its conjugated derivatives) serve as the main germination signals for *C. difficile* spores and promote infection (138–140). Specific secondary bile acids, such as ursodeoxycholic acid, have also been shown to inhibit germination; thus, *C. difficile* spores can sense dysbiosis in the gut through both positive and negative regulation by bile acid signals (141, 142). Beyond spore germination, bile acids also potently inhibit *C. difficile* toxin activity via direct binding (142–148). Inhibition of toxin activity by bile acids can be overcome by increased toxin concentration, suggesting that this mechanism of pathogen suppression may not be as effective against strains with increased toxin production (144). Secondary bile acids also have direct effects on *C. difficile* growth and modulation of the host response to infection (140–142, 146, 149, 150).

Commensal Clostridia species belonging to the *Lachnospiraceae*, *Ruminococcaceae*, and *Clostridiaceae* families have been implicated in bile acid-mediated colonization resistance against *C. difficile* through production of secondary bile acids (138). In particular, the 7α-dehydroxylation activity by the bile acid inducible (*bai*) locus of Clostridia, including *Clostridium scindens,* has been shown to be critical for *C. difficile* inhibition (151–153). Deconjugation of bile acids by BSHs, which is a pre-requisite for secondary bile acid production, can be carried out by a broader diversity of gut commensals, and several BSH-encoding species have been demonstrated to suppress *C. difficile* fitness (140, 154, 155). Together, these data support a model in which depletion of both *bai*- and BSH-encoding organisms by antibiotic treatment reduces colonization resistance and suppression of *C. difficile* virulence.

### Nutrient competition

There is hostile competition for resources and nutritional niches within the GI tract during infection. *C. difficile* exhibits incredible metabolic plasticity and is capable of growth on a variety of nutrient sources. In particular, Stickland fermentation of amino acids is a hallmark of Clostridial metabolism (156). Stickland fermentation involves the paired oxidation and reduction of amino acids, which allows for the use of amino acids as a sole carbon and energy source. *C. difficile* is so well-adapted to growth on amino acids that it preferentially consumes amino acids over sugars in minimal media despite a much lower energy output (157). However, commensal Clostridia are also capable of Stickland fermentation, and the direct competition for fermentable amino acids has been shown to be a critical component of microbiome-mediated suppression of *C. difficile* virulence (158–164). Indeed, byproducts of Stickland fermentation were identified in stools of CDI patients, and the ability of *C. difficile* to undergo Stickland fermentation in the gut distinguished symptomatic patients from those asymptomatically colonized with *C. difficile* (165). In addition, amino acid catabolism genes are upregulated in mouse models of CDI, and mutants of *C. difficile* lacking proline or glycine reductase, enzymes necessary for carrying out Stickland reactions, are attenuated *in vivo* (162, 166–168).

Competition for nutrients outside of amino acids also underlies *C. difficile*-microbiota interactions in the gut. A study by Ferreyra and colleagues found that *C. difficile* converts succinate to butyrate for regeneration of NAD+, which is critical during NAD+-consuming metabolism, such as growth on sorbitol (169). Importantly, consumption of succinate by *C. difficile* increased pathogenesis, indicating that succinate levels in the gut may alter CDI severity (169). Luminal succinate levels increase after antibiotic treatment, suggesting either increases in succinate-producing bacteria or decreases in succinate-consuming bacteria. Subsequent studies demonstrated that succinate-consumers could provide colonization resistance against *C. difficile*, indicating that the latter scenario is likely (170–172). In addition to providing a metabolic advantage to *C. difficile*, succinate has also been shown to increase *C. difficile* biofilm formation, which may help drive persistence in the gut (173). Additionally, mucus-derived sugars such as N-acetylglucosamine and sialic acid have been shown to be important carbon sources for *C. difficile* during infection (174). Commensal microbes which utilize mucus-derived sugars provide colonization resistance against *C. difficile*, indicating that competition for these carbohydrates is an important determinant of CDI susceptibility (175).

### Microbiota-derived inhibitory compounds

In addition to depleting metabolites that *C. difficile* can utilize for growth, the gut microbiome can also produce inhibitory compounds that reduce *C. difficile* fitness. The SCFA butyrate, which is produced by commensal Clostridia in the gut, has been shown to reduce *C. difficile* colonization and disease severity in animal models (176–178). Additionally, reduced butyrogenic microbial taxa and fecal butyrate levels are associated with CDI patients vs healthy or asymptomatic controls (176, 179–181). Several mechanisms of butyrate suppression of *C. difficile* have been identified. Exogenous butyrate slows the growth rate of *C. difficile* by forcing its own endogenous butyrogenic reactions to work in reverse, which is energetically inefficient (176, 182). Butyrate also protects the intestinal barrier against damage during CDI and dampens pathogenic immune responses, which was shown to be dependent on shifts in bile acid metabolism and HIF-1 signaling (181, 183). Interestingly, exogenous butyrate enhances *C. difficile* sporulation and toxin production *in vitro*, suggesting that the beneficial effects of butyrate on the host response and suppression of *C. difficile* growth outweigh the increase in virulence properties (182, 184, 185).

Other inhibitory compounds secreted by the gut microbiome have been identified. The secondary bile acid-producing organisms *C. scindens* and *Paraclostridium sordellii* were found to produce tryptophan-derived antibiotics that prevent *C. difficile* growth, ascribing another function to these species in protecting against CDI outside of bile acid metabolism and nutrient competition (186). The metabolite urolithin A, produced by gut microbes from polyphenols, ameliorates CDI disease severity in mice by reducing *C. difficile* toxin production (187). Interestingly, only 40%–50% of people have urolithin A-producing capacity in their gut microbiome, suggesting that the presence of this pathway may be protective in a subset of patients (187). Reuterin, a natural product of the gut bacterium *Lactobacillus reuteri*, blocks *C. difficile* growth and toxin production through generation of damaging reactive oxygen species (188–190). Other *Lactobacillus* species such as *Lactobacillus gasseri* are also capable of direct inhibition of *C. difficile* growth and promotion of colonization resistance in mice (191). However, this study also found that *Lactobacillus acidophilus* enhanced *C. difficile* colonization, indicating that inhibition is not a common feature of all *Lactobacillus* species (191). The interaction of *C. difficile* and *Lactobacillus* warrants further investigation, as these bacteria are commonly used as over-the-counter probiotics.

Fungi have also been found to have important interactions with *C. difficile. Candida albicans* gut colonization was recently reported to be associated with CDI (192, 193). In mouse models of CDI, *C. albicans* ameliorated CDI severity by inducing protective IL-17 responses and altering gut lipid composition (194, 195). However, one study found the opposite effect, with *C. albicans* increasing disease severity (196). *In vitro*, *C. albicans* is capable of increasing the tolerance of *C. difficile* to oxygen, which may help *C. difficile* persist in the inflamed gut (197). In addition, failure of fecal microbiota transplantation (FMT) therapy was associated with *C. albicans* (193). While the data on what role *C. albicans* plays during CDI are conflicting, *C. difficile*-fungi interactions are an intriguing and underexplored area of future research.

### Synergistic interactions with *C. difficile*

Mechanisms of synergy between the gut microbiome and *C. difficile* are less well understood. Studies on microbial composition of CDI patient stools have found associations between certain bacterial taxa, including *Enterococcus*, *Akkermansia*, *Streptococcus*, *Haemophilus,* and *Enterobacteriaceae*, that correlate with *C. difficile* colonization, expression of toxin, severe disease, and recurrence (179, 198–201). Similar results in mouse models of CDI show increased abundance of *Enterococcus*, *Enterobacteriaceae,* and *Streptococcus* in mice that experienced more severe disease (202–204). We recently found that *Enterococcus* species enhance *C. difficile* toxin production and growth through conversion of arginine to ornithine, an amino acid that *C. difficile* can use as a substrate for Stickland fermentation (205). Additionally, *E. faecalis* alters *C. difficile* gene expression and motility phenotypes through modulation of phase variation on the *C. difficile* chromosome (206). These results provide evidence of direct enhancement of *C. difficile* virulence by members of the gut microbiota and provide a proposed model for why patients with high levels of these *Enterococcus* often have worse outcomes.

*Clostridium innocuum* is a toxin-negative Clostridial species commonly found in the human gut that can cause rare infections following vancomycin treatment in immunocompromised patients (207). Of note, *C. innocuum* is often associated with inflammatory bowel disease and has been reported to have higher rates of colonization in these patients compared to *C. difficile* (208). A recent report found that *C. innocuum* enhances *C. difficile*-dependent epithelial cell killing and barrier damage in *in vitro* models (207). Another commensal Clostridial species, *Clostridium sardiniense*, was also found to increase CDI disease severity in mice through a similar ornithine cross-feeding mechanism as *Enterococcus* (161). Despite the protective role that commensal Clostridia play overall, this work demonstrates the potential of these closely related species to synergize with *C. difficile*.

Other examples of nutrient exchange between commensal microbes and *C. difficile* have been described. Experiments using the model gut symbiont *Bacteroides thetaiotaomicron* demonstrate that *C. difficile* can utilize succinate produced by *B. thetaiotaomicron* to aid its expansion in the gut (169). In addition, *B. thetaiotaomicron* releases sialic acid from mucins, which both *Salmonella typhimurium* and *C. difficile* can use as a carbon source (174). Antibiotic treatment was found to drastically increase the concentration of free sialic acid levels in the gut, suggesting that dysbiosis induced by antibiotics shifts toward a higher abundance of sialidase-encoding bacteria in the gut (174).

### Microbial regulation of immunity

Outside of direct interactions with *C. difficile*, the microbiome can also influence the outcome of infection through regulation of host immune responses (209). Despite the findings that succinate can promote expansion of *C. difficile* in the gut, succinate-producing bacteria can also inhibit *C. difficile* disease severity by expansion of the tuft cell compartment and a protective type 2 immune response (210). Similarly, microbiota-induced IL-25 signaling was found to protect against disease severity in mice (211). The microbiota-derived SCFA acetate signals through free fatty acid receptor 2 on both neutrophils and ILC3s to promote a protective immune response in the colon (212). Lastly, *C. butyricum* reduces disease severity and increases fidaxomicin efficacy in mice, potentially through modulation of intestinal metabolites and subsequent attenuation of an inflammatory response in the colon (171). Together, these studies highlight an important role for commensal microbes in modulating immune responses to CDI.

## EXTERNAL FACTORS IMPACTING GUT ECOLOGY

### Diet and nutrition

As the primary source of nutrients used by host cells and the microbiota, diet plays a critical role in influencing ecological interactions in the gut that alter CDI severity. Dietary fiber has been shown to be protective in mouse models of CDI, affecting multiple aspects of *C. difficile* fitness in the GI tract. Mice fed a low-fiber diet have impaired recovery of commensal Clostridia species after antibiotic treatment, promoting the persistence of primary bile acids in the gut and loss of colonization resistance (213). Dietary supplementation with microbiota-accessible carbohydrates such as inulin increases production of the fermentation end-products acetate and butyrate, which suppress *C. difficile* virulence as previously discussed (176, 177, 214). Finally, low-fiber intake was associated with higher mortality in mice, which was due to increased dissemination of opportunistic pathogens and secondary sepsis (215). Collectively, these data show that fiber plays a clear protective role during CDI and may be an easy clinical intervention in CDI patients to reduce disease severity (216).

In contrast to fiber, dietary sugars and fats enhance *C. difficile* pathogenesis. *C. difficile* consumption of dietary sorbitol and trehalose in the gut increases *C. difficile* colonization and disease severity in mice (94, 217). Interestingly, the epidemic ribotypes RT027 and RT078 have acquired genetic changes that increase trehalose utilization and enhance virulence (217). Genetic analysis of over 900 *C. difficile* strains found evidence of positive selection in sugar utilization genes, suggesting that dietary sugar availability is an important selective pressure for emerging *C. difficile* strains (218). Sucrose, which *C. difficile* cannot metabolize, indirectly enhances disease severity by decreasing taxa that provide colonization resistance and increasing the abundance of *Enterococcus* (219, 220). High-fat diets were shown to increase CDI severity through a similar mechanism of microbiome alteration as sucrose, and a concomitant increase in primary bile acids (221–223). Bile acid dysregulation was compounded by the effect of high fat increasing synthesis of primary bile acids in the liver (221). In addition, a high-fat diet promoted the increase of neutrophilic immune responses and decreased protective immune responses, such as eosinophilia and ILC3 activation (224).

The abundance of free amino acids in the gut is an important determinant of CDI susceptibility, as this fuels pathogen expansion through Stickland metabolism; indeed, mice fed a low protein diet or diets devoid of proline or ornithine were protected against *C. difficile* colonization (162, 225). However, not only does protein concentration matter, but the protein source was also shown to be critical. Yakabe and colleagues fed mice diets with matching nutritional value but protein sourced from either soy protein or casein. Soy protein led to increased intestinal amino acid concentrations and increased disease severity through expansion of *Lactobacillus* species that can break down soy protein into free amino acids (226).

Micronutrients have also been found to play an important role in CDI pathogenesis. Metals are of particular interest during enteric infections, as sequestration of metals in the gut by proteins such as calprotectin and lactoferrin is a major form of pathogen exclusion known as nutritional immunity (227). Excess dietary zinc overcomes calprotectin-mediated zinc limitation during CDI, leading to elevated tissue zinc levels, expansion of *Enterococcus*, and increased disease severity (228). In contrast, mice fed a high-iron diet experienced a near complete attenuation of disease, associated with reduced neutrophil influx and inflammatory markers, reduction of intestinal proline, and increased secondary bile acids (229). Overall, metals have profound effects on the microbiome and host response, and more work on dietary metals and CDI is warranted.

### Pharmaceutical drugs

Certain pharmaceutical drugs can exacerbate the severity of CDI through modulation of gut ecology. Both acute and chronic use of nonsteroidal anti-inflammatory drugs (NSAIDs), which inhibit cyclooxygenase enzymes and reduce the production of eicosanoids, are associated with an increased risk of CDI (230–232). Studies in mouse models have shown that NSAIDs exacerbate CDI severity through several potential mechanisms, such as microbiome disruption, epithelial barrier injury, and immune dysregulation (233). In particular, NSAID-induced mitochondrial dysfunction renders intestinal epithelial cells more susceptible to the action of the toxins produced by *C. difficile* (234).

Proton-pump inhibitors (PPIs) affect the gut through reduction of gastric acid production, which has been shown to facilitate bacterial colonization (235). Some large observational studies report a higher risk of CDI in PPI users, compared to H2 receptor antagonist users (236, 237). However, recent studies that considered competing factors, such as antibiotic exposure and patient comorbidities, found no significant increase in CDI among patients treated with PPIs (238, 239). More clinical and mechanistic studies are needed to more clearly define the effect of PPIs on susceptibility to CDI.

Recent observational studies and meta-analyses suggest that opioid exposure is associated with an increased risk of CDI in hospitalized patients (240, 241). Clinical studies also indicate that opioid use as an analgesic during CDI correlates with worse outcomes, such as extended hospitalizations and the risk of severe disease (242–244). While the mechanisms of CDI exacerbation by opioids are unknown, possibilities include promoting dysbiosis, suppressing immune function, and reducing gastrointestinal motility; the latter may lead to increased contact time of *C. difficile* with the intestinal mucosa and increased epithelial exposure to bacterial toxins (245, 246). Conducting further studies to better explain the correlation between CDI and opioid use is warranted, as over 70% of CDI patients may be receiving opioid administration during hospital stays (244).

## THE FUTURE OF MICROBIOME AND ECOLOGY-TARGETED CDI THERAPIES

Development of effective therapeutics for CDI has been a significant challenge. Targeting *C. difficile* directly through antibiotic treatment inadvertently kills the closely related commensal Clostridia species that are critical for restoration of colonization resistance and preventing recurring infections. A number of new treatments are in development that aim to treat CDI by manipulating the ecological interactions in the gut to favor the host.

Microbiome-based therapies such as FMTs have been used to treat recurrent CDI (rCDI) for a number of years. FMTs use stool from healthy donors to restore colonization resistance after antibiotic treatment for CDI, with up to 90% efficacy reported (247). As a donor-based therapy, there is substantial variation between donors, as well as the risk of transferring opportunistic pathogens (248). To mitigate these limitations, the development of standardized fecal microbiota products has resulted in FDA approval of two microbiome-based therapies for rCDI, Rebyota, and Vowst (249, 250). Similar donor-derived products in development include RBX7455, a lyophilized oral formulation of Rebyota, MET-2, and CP101 which all had favorable results in initial trials (251–253). Live biotherapeutic products (LBPs) with smaller, defined consortia are also under development and are attractive as a therapeutic option due to their simplicity in manufacturing and known composition. Recent advances in understanding mechanisms of failure for FMTs, including reduced engraftment of specific taxa that produce butyrate, BSHs, and secondary bile acids (199, 254–258), have allowed for the development of defined LBPs that target these specific functions, such as VE303, which uses 8 commensal Clostridia strains, MTC01, which contains 15 obligate anaerobes, or ADS024, which uses only a single *Bacillus velezensis* strain (259–264).

LBPs using non-toxigenic *C. difficile* (NTCD) strains are of significant interest due to their ability to suppress infection by toxigenic *C. difficile* (265–269). Although exact mechanisms are unknown, it is hypothesized that direct competition for nutrients, combined with a reduced metabolic need for the production of large exotoxins, allows NTCD strains to outcompete toxigenic *C. difficile*. Importantly, this suppressive effect is only seen if the NTCD is administered before challenge with toxigenic *C. difficile*, suggesting that dominating *C. difficile* strains in the gut retain a competitive advantage over invading strains (265, 269). The NTCD strain M3 is currently in clinical development as a prophylactic intervention for both primary and rCDI (270–273).

While FMTs, fecal microbiota products, and LBPs are designed to influence the metabolic state of the gut for *C. difficile* exclusion, direct administration of metabolites can have a similar effect. Synthetic bile salt analogs target *C. difficile* germination through multiple mechanisms. Analogs of cholate, taurocholate, ursodeoxycholate, and chenodeoxycholate act as competitive inhibitors of taurocholate-mediated spore germination and can prevent CDI or reduce severity in animal models (274–282). Synthetic bile acid derivatives can also mimic the direct binding and inhibition of TcdB to prevent disease (147). Recently, a metronidazole-ursodeoxycholic acid hybrid was designed to both target vegetative cells and prevent spore germination, which reduced development of rCDI in mice (283).

Modulation of the host immune response can mitigate severe disease by promoting protective pathways and/or suppressing pathogenic responses. Activation of TLR7 with resiquimod (R-848) stimulates IL-22 production by ILC3s to promote epithelial repair and protection from CDI (43). Treatment of mice with the IL-1R antagonist anakinra blocked toxin-induced epithelial injury by ameliorating inflammasome-mediated inflammation (25). Similarly, the prostaglandin E_1_ analog misoprostol reduces CDI severity by attenuation of a pathogenic neutrophil response (284, 285). Other compounds that block neutrophil influx, such as the pregnane X receptor agonist pregnenolone 16α-carbonitrile (PCN), also reduce colonic inflammation and intestinal injury (286). Several of these treatments are currently FDA approved for other indications, which may potentiate their use for CDI in the clinic.

## CONCLUSIONS

CDI is an incredibly complex enteric infection, and susceptibility to and outcomes of infection are shaped by the interplay between *C. difficile*, the host, and the microbiome. While some of these interactions are well understood, such as the manipulation of bile acids, SCFAs, and nutrient pools by commensal microbes, others remain important areas of future research. The balance between interactions which promote and suppress *C. difficile* pathogenesis ultimately decides patient outcome; however, predicting which direction the scale will tip in any given patient remains quite the challenge. Recent advances in systems biology approaches have led to the development of computational models that use data from mouse models and CDI patients to simulate the complex gut environment during infection (87, 287). The use of these models will further our understanding of which interactions supersede the others to push a patient toward susceptibility or survival. Ultimately, understanding the ecological interactions in the GI tract during CDI is critical, as the development of novel therapies to manipulate these interactions shows great promise in ameliorating morbidity and mortality due to CDI.
